# Robotic multidisciplinary endometriosis surgery with multi‐visceral resection: evaluation of short‐term feasibility and safety outcomes

**DOI:** 10.1111/ans.70058

**Published:** 2025-03-05

**Authors:** Joseph Do Woong Choi, Lauren Hofmann, Andrew Craig Lynch, Assad Zahid, Praveen Ravindran, Walid Barto, Yogesh Nikam, Stephen Pillinger

**Affiliations:** ^1^ Australian Robotic Colorectal Surgery Sydney Adventist Hospital Sydney New South Wales Australia; ^2^ Faculty of Medicine and Health The University of Sydney Sydney New South Wales Australia; ^3^ Department of Obstetrics and Gynaecology Sydney Adventist Hospital Sydney New South Wales Australia; ^4^ School of Medicine and Psychology The Australian National University Canberra Australia

**Keywords:** endometriosis, feasibility, multidisciplinary, operative outcomes, robotic surgery, safety

## Abstract

**Background:**

Despite growing interest in robot‐assisted surgery, the literature remains limited on the application of robotic surgery for complex endometriosis surgery requiring multidisciplinary input for multi‐visceral resection. The aim of the study was to report the short‐term feasibility and safety outcomes of this technique from a high‐volume robotic surgery facility.

**Methods:**

This was a single centre, retrospective study evaluating prospectively collected data. All women underwent planned multidisciplinary robotic surgery using the da Vinci Xi system between January 2018 and July 2024. Fifty‐eight patients were included in the analysis of demographic, operative and 30‐day postoperative data.

**Results:**

The median age was 40.5 (range 21–55), with 94.82% of women having ASRM grade 3–4 endometriosis. Almost half of the patients had total hysterectomy and bilateral salpingectomy. Concurrent colorectal resections included appendicectomy/stapled caecectomy (41.38%), rectal shaving (36.21%), rectal wedge resection (39.66%), endo‐anal discoid resection (1.72%) and rectal segmental resection (15.52%). Partial bladder excision and extensive ureterolysis for ureteral stenosis occurred in 5.17% and 11.54%, respectively. The median console time was 148 min (range 49–480 min), 0% conversions and a median 100mls estimated blood loss. Median length of stay was 3 days (range 1–7). Clavien‐Dindo complications ≥2 occurred 5.17% of cases. There were no anastomotic leaks, other infectious complications, postoperative ileus, blood transfusion requirements or mortality within 30 days.

**Conclusions:**

The robotic assisted approach is feasible and safe with overall short operative time, acceptable blood loss, no conversions, relatively short length of stay and minimal short term postoperative complications.

## Introduction

Endometriosis is a chronic inflammatory condition when endometrial‐like tissue are found in extrauterine sites.^1^, ^2^ It is a common condition with approximately 11.4% of women being affected, and those aged 30–34 years most likely to receive the diagnosis.^2^ Five‐12% of women diagnosed with endometriosis will have deep infiltrating endometriosis (DIE), when there is extension of endometriotic deposits under the peritoneal surface of the gastrointestinal system and genitourinary tract.^3^, ^4^, ^5^ Conventional laparoscopy has become the gold standard in the management of DIE since the 2010s, however robotic surgery is emerging as an alternative form of minimally invasive surgery, with limited evidence given its relatively recent introduction.^6^ The complexity of advanced American Society for Reproductive Medicine (ASRM) 3–4 endometriosis in particular, with multiorgan nodules lends itself well to multidisciplinary robotic surgery.^4^ The advantages include greater dexterity with more dynamic range of motion, improved ergonomics, three dimensional (3D) visualization, motional scaling to eliminate tremor, and surgeon control of up to three surgical arms.^7^

Multidisciplinary intraoperative input from gynaecologists, colorectal surgeons and urologists is expected to ensure that an appropriate resection is performed in a safe and timely manner.^4^ Although several recent meta‐analysis have demonstrated non‐inferiority of robotic endometriosis surgery compared to conventional laparoscopy,^3^, ^8^, ^9^ in our experience it has significantly improved identification of endometriotic deposits, with more accurate resection of the disease. The purpose of this pilot study was to evaluate the feasibility and safety of a multidisciplinary approach for complex endometriosis robotic assisted surgery which required concurrent visceral resection for colorectal and/or genitourinary endometriosis.

## Methods

This was a single centre retrospective pilot feasibility study derived from a prospective database performed at a tertiary care hospital. Institutional ethics approval was granted by Adventist HealthCare Limited Human Research Ethics Committee (HREC), study reference number 2024‐022. The patients were identified through the ‘SANCare’ surgical database of all robotic procedures using the da Vinci Xi robotic surgical system (Intuitive surgical, Sunnyvale, CA, USA) performed between June 2018 and July 2024. All proceduralists were high volume robotic surgeons undertaking >50 robotic procedures per year.

The inclusion criteria were: (a) females who underwent robotic endometriosis excision surgery that required concurrent visceral resection, (b) visceral resection defined as at least appendicectomy, caecetomy, colorectal shave/disc excision/segmental bowel resection, bladder excision, extensive ureterolysis for ureteral obstruction from endometriosis or ureteric excision and reimplantation, (c) age > 18 years old and (d) planned multidisciplinary robotic resection (either single or dual console). The exclusion criteria were: (a) robotic excision of endometriosis only, or had concurrent hysterectomy, ovarian cystectomy and/or oophorectomy only, (b) no endometriosis found on formal histopathology and (c) significant missing patient data.

Patient data were extracted from pre‐operative consultation notes, anaesthetic charts, surgical operation reports and histopathology reports. Pre‐operative demographic data was collected including age at surgery, body mass index (BMI), American Society of Anestheologists (ASA) grade, and a description of their pre‐operative symptoms (including dysmenorrhea, dyspareunia, dyschezia, rectal bleeding and infertility). Operative data collected included surgical specialties involved, location of endometriotic nodules, presence of ovarian endometriomas, the type of recto‐sigmoid excision performed, the occurrence of simultaneous hysterectomy, salpingectomy, cystectomy and/or oophorectomy, console time, total surgical room occupancy time, estimated blood loss, intraoperative complication occurrence and the need for conversion from a robotic approach to a laparoscopic or open approach. The operative time also included simultaneous hysteroscopy, cystoscopy, flexible sigmoidoscopy and firefly fluorescence imaging of intravenous/ureteric indocyanine green (ICG) in most patients. Thirty‐day post‐operative data were evaluated for length of hospital stay, anastomotic leak (as defined as ‘a defect of the intestinal wall at the anastomotic site leading to a communication between the intra and extra‐luminal compartments’^10^), post‐operative ileus >3 days, return to theatre, unplanned intensive care unit (ICU) admission and mortality.

Statistical analysis was performed using SPSS software (Statistical Package for Social Sciences for Windows Version 22). Categorical variables were presented as frequency (*n* (%)), and non‐normal distributions reported as median with range or interquartile range (IQR).

## Results

In total, 101 patients were identified as undergoing robotic endometriosis excision surgery, with 58 patients included in the analysis after inclusion and exclusion criteria were applied.

The median age was 40.5, with a BMI of 25. The ASA grade ranged between 1 and 3, with a median of 2. Most women (72.41%) had chronic pelvic pain (more than 3 months) and dysmenorrhea (70.70%) preoperatively. More than 93% of the women had grade 4 endometriosis, and 12.07% of the patients had a history of previous endometriosis surgery (Table 1).

**Table 1:** Patient demographics and characteristics

Patient characteristics	Cohort (*n* = 58)
Age, median, range	40.5 (21–55)
BMI, median (IQR)	25 (22–27.83)
ASA grade, median, range	2 (1–3)
Indication for Surgery (%)	
Chronic pelvic pain	72.41%
Dysmenorrhoea	70.70%
Dysparenuria	65.51%
Abnormal uterine bleeding	41.38%
Dyschezia	39.66%
Infertility	27.59%
Regular use of laxatives	25.86%
Haematochezia	15.52%
Grade of endometriosis (%)	
4	93.10%
3	1.72%
2	1.72%
1	3.46%
History of endometriosis surgery (%)	12.07%

Abbreviations: ASA, american society of anestheologists; BMI, body mass index.

All procedures had multidisciplinary input involving gynaecologist, colorectal surgeon and/or urological surgeon. Most patients had obliteration of Pouch of Douglas (87.94%) and required adhesiolysis of the rectovaginal space (94.83%). Almost half of the women had total hysterectomy and bilateral salpingectomy. About a third of the patients had a unilateral ovarian cystectomy due to concurrent endometrioma (31.03%). Appendicectomy or stapled caecectomy occurred in 41.38% of cases. Rectal shaving and rectal wedge resection with a robotic stapler occurred in 36.21% and 39.66% of the cases respectively. There was a relatively low rate of rectal segmental resection with primary anastomosis in 15.52% of the cases, and 1.72% discoid excision using an endo‐anal circular stapler. A urologist was involved for ureterolysis for ureteral stenosis (11.54%) and partial bladder resection (5.17%). The median operative (console) time was 148 min (range 49–480 min), median total surgical room occupancy time of 254 min (111–590 min) and 100 mL median EBL. There were no conversions to laparoscopic or open surgery. The median histologically confirmed endometriosis excised was 5 nodules (Table 2).

**Table 2:** Procedural characteristics

Procedural characteristics	Cohort (*n* = 58)
Total hysterectomy (%)	46.55%
Salpingectomy (%)	
Unilateral	8.62%
Bilateral	44.83%
Oophrectomy (%)	
Unilateral	27.59%
Bilateral	1.72%
Ovarian cystectomy (%)	
Unilateral	31.03%
Bilateral	6.90%
Rectal shaving (%)	36.21%
Rectal wedge resection with stapler (%)	39.66%
Discoid resection (%)	1.72%
Rectal segmental resection (%)	15.52%
Appendicectomy or caecectomy (%)	41.38%
Stoma formation (%)	1.72%
Partial bladder resection (%)	5.17%
Ureterolysis for ureteral stenosis (%)	11.54%
Obliteration of POD (%)	87.94%
Rectovaginal adhesiolysis (%)	94.83%
Vaginal infiltration (%)	22.41%
Console time minutes, median, range	148 (49–480)
Total surgical room occupancy time, median, range	254 (111–590)
Conversion to laparoscopic or open (%)	0%
Estimated blood loss mls, median (IQR)	100 mL (50–200)
Number of endometriosis nodules excised, median (IQR)	5 (4–7)
Planned multidisciplinary procedure (%)	100%

Abbreviations: IQR, interquartile range; POD, pouch of douglas.

Postoperatively, 6.90% of the patients had Clavien‐Dindo 1 complications within 30 days relating to readmissions for postoperative pain requiring oral analgesics, and per vaginal (PV) bleeding requiring observation without intervention. Clavien‐Dindo 2 and 3 complications occurred in 5.17% of patients due to respiratory distress from pneumoperitoneum related subcutaneous emphysema requiring 1 day in ICU with noninvasive ventilation, PV and per rectal (PR) bleeding requiring tranexaminc acid in another patient, and another requiring takeback for ureteral dilatation and exchange of ureteric stent for ongoing hydronephrosis within 30 days. There were no anastomotic leak or other infectious complications, ileus, need for blood transfusion, or mortality within 30 days. The median length of stay was 3 days (range 1–7 days) (Table 3).

**Table 3:** Postoperative characteristics

Postoperative characteristics	Cohort (*n* = 58)
Length of inpatient stay days, median, range	3 (1–7)
Clavien–Dindo classification <30 days (%)	
1	6.90%
2	3.45%
3	1.72%
4	0%
Anastomotic leak <30 days (%)	0%
Postoperative ileus <30 days (%)	0%
Ureteral trauma/fistula <30 days (%)	0%
Postoperative blood transfusion <30 days (%)	0%
Pelvic haematoma or abscess <30 days (%)	0%
Rectal bleeding requiring endoscopic treatment <30 days (%)	0%
Takeback <30 days (%)	1.72%
ICU admission <30 days (%)	1.72%
Mortality <30 days (%)	0%

Abbreviation: ICU, intensive care unit.

## Discussion

The results of this study indicate the short‐term feasibility and safety of multidisciplinary robotic surgery for endometriosis requiring concurrent visceral resection. There were no conversions to laparoscopic or open surgery, small EBL, relatively short console time, minimal postoperative complications and short inpatient stay. Almost 95% of the patients had ASRM grade 3–4 endometriosis, indicating that robotic assisted surgery can be appropriately utilized for complex cases.

Robotic surgery offers several benefits including enhanced dexterity, advanced imaging, wrist‐like articulation of instruments and ergonomic access to deep and narrow anatomical compartments such as the pelvis. It reduces surgeons physical burden, allowing for sustained concentration and reduced fatigue during complex endometriosis surgeries.^11^ Additionally, 3D robotic visualization was associated with 2.36 times the likelihood of detecting a confirmed endometriosis lesion compared to 2D vision from conventional laparoscopy.^12^ These features allow the potential for robotic surgery to enhance surgical outcomes, reduce recovery times and improve the safety and efficacy of complex procedures especially in advanced endometriosis.^13^

One of the challenges of robotic surgery is loss of tactile feedback, which may limit the differentiation of fibrotic tissues in endometriosis. However, with over 5000 collective robotic procedures performed in our institution and more than 3000 cumulative robotic experience by the authors in general, our experience is consistent with other experienced units in that the cerebrum learns other feedback cues to compensate. The fifth generation Da Vinci system introduced in 2024 has incorporated force feedback features which may overcome haptic limitations for some surgeons.^14^ In addition, the incorporation of near‐infrared fluorescence imaging with indocyanine green coupled with artificial intelligence may further enhance endometriosis detection and excision.^15^, ^16^ Another drawback of robotic surgery is that the high initial acquisition costs limit accessibility of robotic surgery.^13^ As such, robotic systems are generally only available in private hospitals and are very limited in public hospitals in Australia. Robotic surgery for DIE involving the digestive tract was associated with higher cost for colorectal resection (+352.6 euros, +14%), rectal disc excision(+378 euros, +25%) compared to conventional laparoscopy.^17^ Efforts are ongoing to make robotic surgery more cost‐effective and widely available.

Most of the current evidence is limited to retrospective studies with heterogeneous disease severity, and comparison of robotic surgery with outcomes compared from established laparoscopic surgeons. The first significant publication reporting a single arm series of robot assisted surgery for stage 4 endometriosis was by Collinet *et al*., who demonstrated the feasibility of the procedure with a mean operative time of 180 min, which was slightly longer than our study (148 min).^18^ This was followed by a comparative analysis of robotic versus laparoscopic surgery for grade 3–4 endometriosis by Nezhat *et al*., who concluded that the robotic platform was associated with increased operative time (196 versus 135 min) and increased hospital stay (day 1 versus day 0).^19^ However, this study excluded patients who required bladder, ureteral, bowel resection or hysterectomy. LACROSE is the only current randomized controlled trial that compared 35 robotic versus 38 laparoscopic patients for endometriosis surgery.^20^ They included grade 1–2 and 3–4 endometriosis and excluded concomitant bowel resection and/or ureteral re‐anastomosis patients. The two techniques were found to be equivalent in regard to operative time (106.6 versus 101.6 min), blood loss (100.9 versus 43.8 mL), complications or rates of conversion to laparotomy (0 versus 2.6%).^20^ However, these results did not focus on the more complex cases where the contribution of robotic surgery is thought to be augmented.

Verrelli *et al*. published results on robotic versus laparoscopic surgery for severe endometriosis, which included patients who had concurrent surgery for digestive tract (requiring rectal shaving, discoid resection and segmental resection), bladder, sacral plexus and diaphragm involvement.^17^ There was statistically higher total surgical room occupancy (203 versus 151 min) and operating time (150 min versus 105 min) in the robot group without differences in mean hospital stay and postoperative complications.^17^ These results were partly echoed in a recent meta‐analysis that found a significantly longer operative time and longer hospital stay in the robotic group, without significant differences in intra‐ or postoperative complications, conversion rate or blood loss.^3^

Deep infiltrating endometriosis of the bowel occurs in 5.2%–12% of women with endometriosis, with the rectum, rectosigmoid and appendix commonly affected.^4^, ^21^, ^22^ A prospective study on colorectal endometriosis found that robotic surgery was an adequate alternative to laparoscopy when they reported a higher surgical room occupancy time (281 versus 208 min) and operating time (221 versus 163 min) in the robotic group, without differences in blood loss, complications rates, voiding dysfunction and mean hospital stay (8 versus 6.5 days). There were also no increased risk of main postoperative complications for excision of large >3 cm rectal endometriosis nodule in the robotic group.^23^ Another prospective study echoed the longer operative time in the robotic group, however they also reported higher rates of healthy margins, and a lower intraoperative complication rate with a discoid or segmental resection compared to conventional laparoscopy.^24^

Urinary tract endometriosis occurs in 0.3%–12% of patients with pelvic endometriosis.^25^ The bladder is involved in 80% of cases, and ureteral endometriosis in 14% of cases.^5^, ^26^ There are no prospective studies comparing robotic and laparoscopic/open surgery for urinary tract endometriosis. Di Maida *et al*. retrospectively reported 46 patients who underwent robotic partial cystectomy, ureteral reimplantation, ureterolysis and/or excision of superficial bladder nodule.^27^ They concluded that robotic excision of urological endometriosis was a safe and effective treatment option, with a low (10.9%) complication rate,^27^ consistent with findings of Giannini *et al*.^28^ A recent systematic review found that the for bladder endometriosis, robotic surgery was feasible and associated with reduced postoperative pain, shorter hospital stays and faster recovery.^29^

The study is not without limitations. First, it was a single arm, non‐comparative retrospective study using a prospective database with potential for selection bias. Second, although the cohort was mostly ASRM grade 3–4 endometriosis (~95%) favouring complex cases, the severity of the disease was nonetheless heterogeneous, as was the type and indication of operation. As such, the results may not be specific to a particular type of cohort. Third, the histopathology did not report on the excision margins which limited the completeness of endometriosis excision. Finally, we did not report on cost, or long‐term operative and quality of life outcomes following robotic endometriosis surgery due to incomplete data. It is the authors' intention to report on these variables in a future study.

## Conclusions

This pilot study has demonstrated the short‐term feasibility and safety of robotic assisted multidisciplinary endometriosis surgery with concurrent visceral resection. Overall, the operative time was shorter than the quoted literature, with no conversions to laparoscopic or open surgery, acceptable blood loss, minimal complications and relatively short inpatient stay. Increasing experience and future studies reporting functional and long‐term surgical outcomes, cost, comparison with conventional laparoscopy and results of other studies including the ROBEndo trial^30^ (scheduled to complete in 2026) is expected to further advance the literature in robotic multivisceral endometriosis surgery.

## Author contrisbutions

**Joseph Do Woong Choi**: Data curation, formal analysis, investigation, methodology, project administration, writing—original draft, writing—review and editing. **Lauren Hofmann**: Data curation, formal analysis, investigation, methodology, writing—original draft, writing—review and editing. **Andrew Craig Lynch**: Formal analysis, supervision, validation, writing—review and editing. **Assad Zahid**: Formal analysis, supervision, validation, writing—review and editing. **Praveen Ravindran**: Formal analysis, supervision, validation, writing—review and editing. **Walid Barto**: Formal analysis, methodology, writing—review and editing. **Yogesh Nikam**: Conceptualization, formal analysis, investigation, validation, writing—review and editing. **Stephen Pillinger**: Conceptualization, formal analysis, investigation, methodology, supervision, validation, writing—review and editing.

## Funding information

The authors do not have any funding/grant sources to declare for this manuscript.

## Conflict of interest

None declared.

## Ethics approval

Institutional ethics approval was granted by Adventist HealthCare Limited Human Research Ethics Committee (HREC), study reference number 2024–022. All authors declare no other conflicts of interest, or declarations.
